# Syphilis by Conception

**Published:** 1889-04

**Authors:** A. Fournier, A. Dagenais


### *SYPHILIS BY CONCEPTION.*

By Prof. A. FOURNIER, M. D.,

Translated by A. DAGENAIS, M. D , from *L'Abeille Médicale.*

Professor Fournier (in *L'Union Médicale*), whose teaching is always as clear as useful, took this important question, syphilis by conception, at his clinical lessons. It is a mode of contamination by syphilis, which presents a practical interest so much greater that we should derive important prophylactic precaution. Young women recently married are especially the ones who suffer from syphilis in this manner. It generally happens thus: A young girl, pure and sound, marries a man who has already had syphilis incompletely treated. She becomes *enciente*, and soon presents the manifest accidents of syphilis—specific roseola, mucous plaques, headache, alopecia. The consulted physician looks for the initial accident, the chancre. In spite of a minute and complete examination, he finds none. Not only the chancre cannot be discovered, but its habitual companion, the bubo, does not exist. There is not even any adenopathic syphilitic vestiges. In spite of these facts, the woman is manifestly syphilitic. The physician, astonished at not finding the chancre, expects that he will find the lesion which caused the infection on the husband. New surprise: the examination of the husband reveals no recent specific manifestations. The husband, being told of the dangers of a possible contamination, affirms that since his marriage he has watched himself attentively, and that he has not perceived the least redness or the slightest erosion. The complete examination of the subject is negative.

Thus, we have a woman who took syphilis from a man having present no symptoms capable of giving syphilis. It is a complete derogation to the usual laws of syphilis. If these facts were exceptional, we should dismiss them as doubtful, but they are met frequently enough, not in the hospital, but in city practice where they are better observed, and are easily followed up when we are in the presence of such a case; and when we interrogate the woman, we learn that she is either pregnant, that she has been pregnant, or has just had a miscarriage. It is then legitimate to suppose that she has been infected by the fetus. This supposition is further strengthened, as the child is born syphilitic. So, in cases of this nature, the destiny of this child is lamentable. From twenty-two cases of syphilis by conception, observed by Diday, fifteen times the pregnancy was interrupted by a miscarriage, seven times the fetus was born at full time, or nearly so, but was either dead, or died soon after birth, showing syphilitic manifestations. These figures are confirmed by Professor Fournier's statistics.

The syphilis of the child is then constant; as the mother's, it is an abnormal syphilis without primary period. The woman did not get it from her husband or another man, for we conceive that, with her, contamination has taken place by the immediate contact during several weeks with a syphilitic subject, the fetus. Nothing astonishing that a woman should be affected by that center of syphilis that she carries in her abdomen.

Let us see, now, whether this theory of the infection of the mother by the fetus during its intra-uterine life, is in contradiction with the recent researches on the passage of microbes through the placenta. Let us find out if the theory of this sudden attack of syphilis, due to the possible transmission through the placenta, is not, on the contrary, supported by the new informations of science on infectious diseases.

At first we know that a number of diseases are transmitted from the mother to the child through the placenta, as variola, measles and scarlet fever. Amongst the diseases of microbes well demonstrated, which enter into this list, we must mention acute septicemia and experimental chicken cholera.

It was thought, a few years ago, that the placenta constituted a perfect filter, which sifted all pathogenic microbes; but it is not so.

The remarkable experiments of Strauss and Chámberland have demonstrated that the placenta let certain microbes pass. They have demonstrated that the bacteria of anthrax (charbon) could be found in the blood of the fetus. We then admit that certain diseases are transmissible from the mother to the child through the placenta.

Syphilis has long ago given a clinical proof. So a sound woman becomes pregnant from a sound man—indemnity of syphilis. The child borne is syphilitic. It is then certain that it is through the intermediary of the placenta that the fetus received syphilis from its mother. We readily admit these phenomena of exosmose concerning the placenta. Why not admit the reverse current or infection by endosmose, if, by the placenta, syphilis may be transmitted from the mother to the fetus; why, inversely, the same infection could not be transmitted from the child to the mother?

There is one more reason which pleads in favor of the possibility of the transmission of the syphilis from the fetus to the mother by the placenta. It is that the mother's syphilis presents then the same form as the syphilis of the fetus, which, in its turn, got it from its mother. In both cases, it is a syphilis decapitated, without initial accident, without primary period—"*une syphilis generale d'emblée.*" This similarity of form of the two syphilists confirm the identity of the mode ot introduction of the virulent agent. Bacteriology and experimental pathology are in favor of this theory of syphilis through conception.

We know, to-day, that the effects of viruses differ greatly; that they give rise to symptoms very different, according to their mode of penetration. For example, the charbon (anthrax), if it is inoculated on the skin of the animal, will determine a true charbon—terrible, mortal. If the injection is made in the blood, instead of reproducing the charbon, we, on the contrary, establish immunity by the *vaccination charbonneuse.* Does it not result from these experiments, which have not been contradicted, that similar effects produced by the same virus, under different circumstances, duplicate identity of penetration. When a fetus, outside of all paternal influence, contracts syphilis in utero, which has made its appearance on the mother only after her conception, it is then affected by the maternal blood which came in the placenta. Is it not then logical to suppose that when a woman presents, during her pregnancy, specific accidents, without initial chancre, that she owes this syphilis to a mode of especial contamination, and that, in one word, it is syphilis through sanguine contamination?

Besides, the mother's infection by the child imposes itself by the absence of all other causes which could explain the infection. Then, as we cannot incriminate directly either the husband or any other male factor, nor any external integumentary contagion, is it not more logical to incriminate the fetus, which is syphilitic, and which almost always dies from it? Is it not, then, an occulent center of infection which the woman carries in herself, in her flesh, in her substance? So, according to clinical observations upon bacteriology and pathological physiology, we must admit that there exists for the woman a mode of special contamination—a sound woman conceiving a syphilitic child from a syphilitic man, may be infected through her own child. So may be formulated this variety of syphilitic contamination called conceptional syphilis.

Professor Diday gave the name to the disease. He is the first who studied and described this morbid entity. There is, to-day, hardly any doubt upon the reality of these facts, in spite of the objections which have been brought out.

The principal objection of authors who deny syphilis through conception, consists in saying that this variety of contamination is a fanciful creation, invented for want of anything better; that it is committing a double material error to admit a syphilis without primary period, and admits that, in such a case, the husband was sound. If no chancre has been found, it is because it has been unheeded; it was probably minute, small, ephemeral; it was, perhaps, in an unusual region, where it was difficult to reach (chancre of the vagina, of the cervix, etc.); besides, do we not know that these characters of the chancre are often met with in a woman, and that there is nothing astonishing if we failed to discover the initial accident?

Moreover, when we affirm that the husband did not present any contagious accident when the fecunding coition took place, what proof have we of this immunity of the husband? This man, supposing he was of good faith, can he not be deceived? A minim lesion, insignificant in appearance, has probably escaped his observation. Strictly speaking, in admitting that this man was sound, why is it not a question of syphilis transmitted by the blood, occasioned by an erosion or an excoriation which took place in both persons during the coition? Syphilis through conception does not exist; it is a chimera. It is easy to answer these objections. If cases of this order presented themselves only once in a while, if they were isolated facts, we could oppose them to the end, or not entertain them at all. But these facts are commonly observed, and Professor Fournier has collected personally more than fifty authentic observations. Certainly, once in a while, a chancre may have passed unnoticed, but it is illogical to admit that all authors who have reported such observations were mistaken, or have committed an error of observation. Why would the chancre be always unrecognized only in the case where a woman has become pregnant? To pretend that these observations are bad, or to refuse to accept them, is going against logic or good sense.

In regard to challenging the testimony of the husband, certainly we must be reserved in the appreciation of his account; but we must not be too incredulous when we happen to meet with intelligent persons, of good faith, who confess they had syphilis, but who, being told of the possible danger of a contamination, have observed themselves anxiously before and after each coition. They are rather more disposed to be alarmed uselessly than rest in a deceiving quietude. Besides, in certain observations, the husband was a physician. We can scarcely put in doubt such information.

Finally, there exist, amongst the cases of syphilis through conception, unexceptional observations which challenge any critic. Such is the case reported by M. Gailleton. A young girl, sixteen years old, virgin, became pregnant following one coition. The young man, the ravisher of this virginity, had syphilis six months previously; but, for a month, no symptoms whatever were present. Frightened at the possible consequences, he came the next day to consult M. Gailleton, who examined him thoroughly, but could not discover any specific lesion, and reassured the imprudent youth. But the young girl, who became pregnant, presented, two months afterward, the following symptoms: violent headaches, roseola, mucous plaques; in a word, a syphilis clearly characterized, and without a trace of chancre. Before such facts, doubt is not possible, and we may say that syphilis through conception takes its place in medical science.

As to the mechanism by which the infection is transmitted from the fetus to the mother, it is a problem which has not been solved as yet. Is the infection transmitted by the ovum when in the tube, or after it reaches the uterus? Or, does not infection take place later, through the placenta, after the ovum is grafted to the uterine cavity? There are many suppositions, which would bring us into discussion without end.

